# The association between substance P and white matter integrity in medication-naive patients with major depressive disorder

**DOI:** 10.1038/s41598-017-10100-y

**Published:** 2017-08-29

**Authors:** Eunsoo Won, June Kang, Sunyoung Choi, Aram Kim, Kyu-Man Han, Ho-Kyoung Yoon, Su-Hee Cho, Woo-Suk Tae, Min-Soo Lee, Sook-Haeng Joe, Yong-Ku Kim, Byung-Joo Ham

**Affiliations:** 1 Department of Psychiatry, Korea University College of Medicine, Seoul, Republic of Korea; 2 Department of Biomedical Science, Korea University, Seoul, Republic of Korea; 3 Department of Brain and Cognitive Engineering, Korea University, Seoul, Republic of Korea; 4 Brain Convergence Research Center, Anam Hospital, Korea University Medical Center, Seoul, Republic of Korea

## Abstract

Substance P (SP) has been implicated in major depressive disorder (MDD), with SP antagonists being studied as potential antidepressants. Although impaired neural plasticity is considered a key mechanism in MDD pathophysiology, the association between SP and brain structural changes in depression has not been investigated. We investigated the correlations between SP levels and white matter (WM) integrity in 42 medication-naive patients with MDD and 57 healthy controls (HCs). Plasma levels of SP were determined, and diffusion tensor imaging (DTI) was performed to investigate microstructural changes in WM tracts. In patients, negative correlations between SP levels and fractional anisotropy (FA) values of the forceps minor of the corpus callosum, and positive correlations between SP levels and radial diffusivity (RD) and mean diffusivity (MD) values of the right corticospinal tract (CST) were observed, with no significant correlations in HCs. Linear regression analyses showed SP levels to significantly predict FA values of the forceps minor, and RD and MD values of the right CST in patients, but not in HCs. We consider our findings to contribute to the neurobiological evidence on the association between SP and brain structural changes in depression, which may be related with the pathophysiology and treatment of MDD.

## Introduction

Imbalances in monoamine neurotransmission, mainly serotonin and norepinephrine, have been considered key mechanisms in the pathophysiology of major depressive disorder (MDD), and currently available antidepressants mostly affect monoaminergic transmission accordingly^1^. However, the monoamine hypothesis of depression is suggested to have major limitations, and additional mechanisms are being proposed in the pursuit for novel treatment options^2^. For instance, substance P (SP) has been suggested to play a role in the etiology of MDD, and SP antagonists have been previously studied for their potential antidepressant effects^3, 4^. SP is a neuropeptide that is colocalized with monoamines, and is one of the most abundant neurokinin peptides in the central nervous system that acts as a neurotransmitter or neuromodulator^5^. SP has been implicated not only in the regulation of nociception and pain^6^, but also in the modulation of stress and depression^4^. SP is released as a part of the response to stress and has been suggested to influence physiological and behavioral stress responses^7^. Previous studies have reported exposure to a variety of stressors can cause increases in SP efflux, which is a direct marker of SP neurotransmission, in brain regions implicated in stress reactions^8^. Also, substance P antagonism has been shown to inhibit responses to stressful stimuli^4^. Increased levels of serum and cerebrospinal fluid SP levels were reported in patients with MDD^9, 10^, and repeated administration of antidepressants has been shown to lead to decreased synthesis of SP in certain brain regions^11^. Furthermore, SP shows high affinity to neurokinin 1 (NK1) receptors, which are expressed in a wide variety of brain regions that are implicated in the regulation of emotion, including the hippocampus, amygdala, prefrontal cortex and ventral striatum^12^. Previous studies have reported the antidepressant effects of NK1 receptor antagonists^3, 4^. However, subsequent investigations failed to replicate such positive findings^13^, and the exact mechanism on how SP contributes to MDD is still unknown. Also, although impaired neural plasticity and neurogenesis are considered key mechanisms in the pathophysiology of MDD^14^, the association between SP and brain structural changes in depression has not been previously investigated.

Imaging techniques associated with diffusion tensor imaging (DTI) have made it possible to investigate alterations in microstructural integrity of white matter (WM) tracts^15^. WM integrity has been reported to be sensitive to stress by numerous studies^16^, and decreased integrity of various WM tracts have repeatedly been associated with depression^17^. Poor response to antidepressant treatment has also been linked to decreased WM integrity in patients with MDD^18^. Furthermore, antidepressants have been suggested to mediate their effects by increasing neurogenesis and modulating the signaling pathways involved in plasticity and survival^19^. However, although SP has repeatedly been associated with stress and depression, and the antidepressant effects of SP antagonists are still controversial, no studies have yet investigated the association between SP and WM integrity in MDD. Therefore, in this study we investigated whether SP levels significantly influenced WM integrity in patients with MDD and healthy controls (HCs). First, we hypothesized that patients with MDD would exhibit altered integrity in white matter tracts that are related to cortico-limbic circuit alterations associated with depression, compared to HCs. Second, we hypothesized that SP levels will show negative correlations with WM integrity, and that SP levels will show significant effects on WM integrity in the patient group.

## Results

### Demographic and clinical characteristics, SP levels, DTI parameters

There were no significant differences in the demographic variables tested between patients with MDD and HCs. Significant difference was observed for HDRS scores between diagnostic groups, with mean (standard deviation) HDRS scores being 19.60 (5.78) and 2.00 (2.07) for patients and HCs respectively (p < 0.001). No significant difference in SP levels were detected between the two diagnostic groups (Table 1). For FA values, patients with MDD had lower values in the forceps major of the corpus callosum (F_(1,94)_ = 26.662, p < 0.001) and left inferior longitudinal fasciculus (ILF) (F_(1,94)_ = 14.371, p < 0.001), indicating decreased integrity of the forceps major and left ILF in the patient group compared to the control group. For AD values, patients with MDD exhibited lower values in the left superior longitudinal fasciculus-parietal bundle (SLFp) (F_(1,94)_ = 12.287, p = 0.001) (Tables 2 and S1), indicating decreased integrity of the left SLFp in the patient group compared to the control group.

**Table 1:** Demographic and clinical characteristics and SP levels of medication-naiive patients with MDD and HCs.

	Patients with MDD (n = 42)	HCs (n = 57)	p
Age	41.29 (11.49)	38.44 (13.12)	0.254
Gender (male/female)	11/31	20/37	0.387
Education level			0.320
Elementary and middle school	7	5	
High school or college/university	32	44	
Above graduate school	3	8	
HDRS-17 score	19.60 (5.78)	2.00 (2.07)	**<0.001** ^**a**^
Duration of illness (months)	10.57 (22.66)		
SP level	151.04 (78.31)	162.20 (66.68)	0.526, F_(1,95)_ = 0.405

Data are mean (standard deviation) in age, HDRS-17 scores, duration of illness, and SP levels.

The p values for comparison in age and HDRS-17 scores were obtained by 2-sample t-tests.

The p values for distribution of gender and education level were obtained by chi-square test.

The p values for comparison in SP levels were obtained by analysis of covariance, with age and gender included as covariates.

^a^Significance level p < 0.05.

SP, substance p; MDD, major depressive disorder; HCs, healthy controls; HDRS-17, 17-item Hamilton Depression Rating Scale.

**Table 2:** WM tracts showing significant difference in FA and AD values between medication-naiive patients with MDD and HCs.

WM tracts	Patients with MDD	HCs	F	p
Forceps major, FA	0.527 (0.0957)	0.606 (0.0494)	26.662	<**0.001** ^a^
left ILF, FA	0.465 (0.0471)	0.497 (0.0320)	14.371	<**0.001** ^a^
left SLFp, AD	0.00116 (0.0000579)	0.00119 (0.0000472)	12.287	**0.001** ^a^

Data are mean (standard deviation).

F and P values were obtained using analysis of covariance, adjusted for age, gender and total intracranial cavity volume as covariates.

^a^Bonferroni correction was applied for the 18 WM tracts: 18 comparisons in both hemispheres, p < 0.00278 (0.05/18).

WM, white matter; FA, fractional anisotropy; AD, axial diffusivity; MDD, major depressive disorder; HCs, healthy controls; ILF, inferior longitudinal fasciculus; SLFp, superior longitudinal fasciculus–parietal terminations.

### Correlations between SP levels and DTI parameters

In patients with MDD, negative correlations were observed between SP levels and FA values of the forceps minor of the corpus callosum (Pearson correlation: r = −0.504, p = 0.001), which indicates that as SP levels increase, the integrity of the forceps minor decreases in the patient group. Also, positive correlations were observed between SP levels and RD and MD values of the right corticospinal tract (CST) (RD: Pearson correlation: r = 0.486, p = 0.002; MD: Pearson correlation: r = 0.496, p = 0.001) (Tables 3 and S2), which indicates that as SP levels increase, the integrity of the right CST decreases in the patient group. In HCs, no significant correlations between SP levels and DTI parameters were observed (Supplementary Table S3).

**Table 3:** WM tracts showing significant correlations between SP levels and FA, RD and MD values in medication-naiive patients with MDD.

	WM tracts	FA	AD	RD	MD
SP levels	forceps minor	**−0.504 (0.001)** ^**a**^	−0.044 (0.789)	0.379 (0.017)	0.298 (0.065)
	right CST	−0.311 (0.054)	0.442 (0.005)	**0.486 (0.002)** ^a^	**0.496 (0.001)** ^a^

All data are given as coefficient of Pearson correlation controlling for age, gender and total intracranial cavity volume (p value).

^a^Bonferroni correction was applied for the 18 WM tracts: 18 comparisons in both hemispheres, p < 0.00278 (0.05/18).

WM, white matter; SP, substance p; FA, fractional anisotropy; AD, axial diffusivity; RD, radial diffusivity; MD, mean diffusivity; MDD, major depressive disorder; CST, corticospinal tract.

### Effect of SP levels on WM integrity

**Figure 1:**
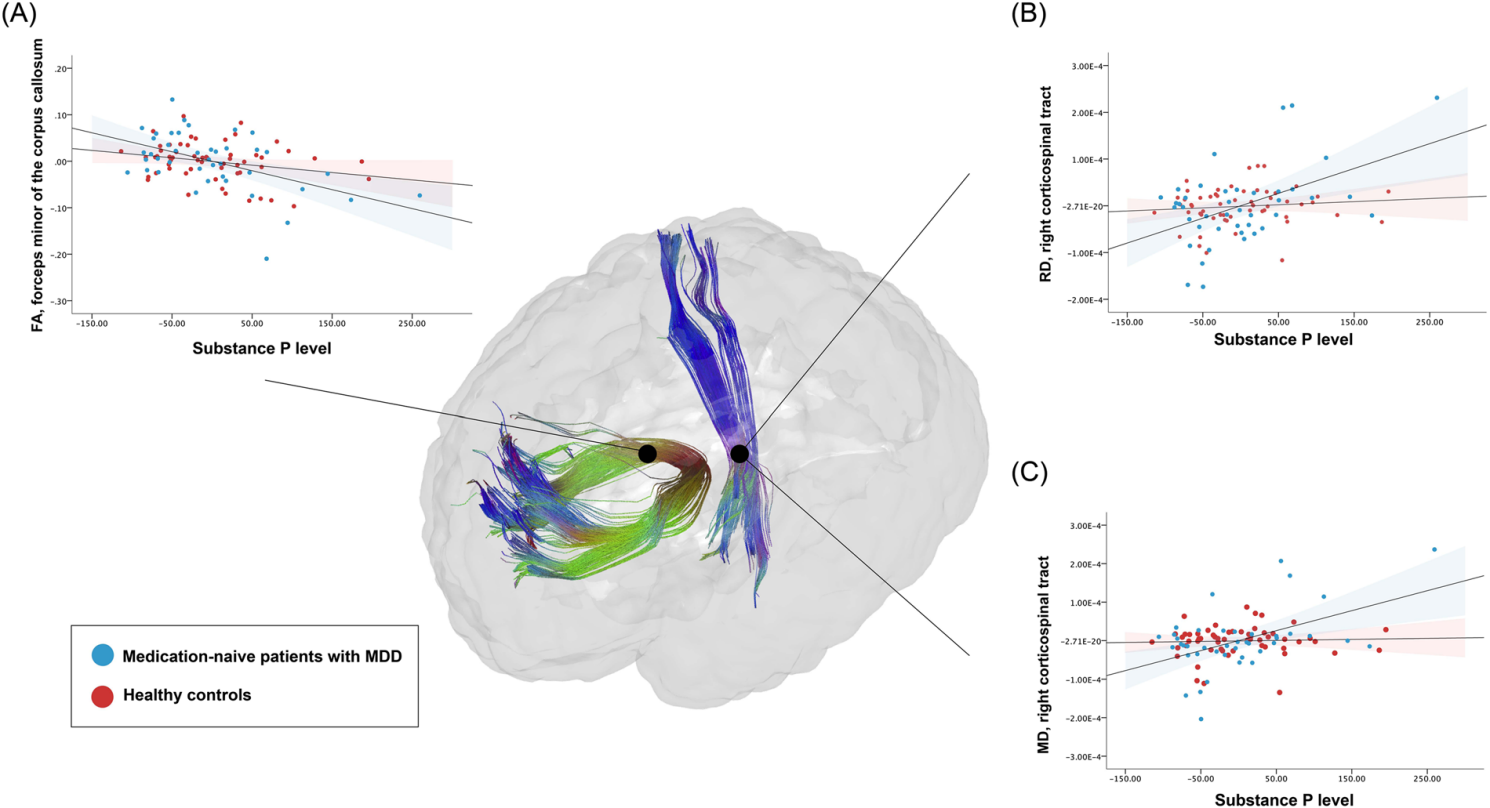
Scatter plots of the linear regression analyses showing the effects of substance P (SP) levels on the forceps minor of the corpus callosum and right corticospinal tract (CST) in medication-naive patients with major depressive disorder (MDD) and healthy controls (HCs). (**A**) SP levels significantly predicted mean FA values of the forceps minor in patients with MDD but not in HCs, which indicates SP levels to have significant influence on the integrity of the forceps minor in the patient group, but not in the control group. (**B**) SP levels significantly predicted mean RD values of the right CST in patients with MDD but not in HCs, which indicates SP levels to have significant influence on the integrity of the right CST in the patient group but not in the control group. (**C**) SP levels significantly predicted mean MD values of the right CST in patients with MDD but not in HCs, which indicates SP levels to have significant influence on the integrity of the right CST in the patient group, but not in the control group.

Table 4 shows the results of the separate multiple regression models with SP level as a predictor and DTI scalar values as outcomes. Among MDD patients, SP level significantly predicted mean FA values of the forceps minor (model: R^2^ = 0.275, p = 0.016; variable: R^2^ = 0.247, β = −0.505, p = 0.001), and mean RD (model: R^2^ = 0.290, p = 0.011; variable: R^2^ = 0.220, β = 0.476, p = 0.002) and MD values (model: R^2^ = 0.286, p = 0.012; variable: R^2^ = 0.233, β = 0.490, p = 0.001) of the right CST. This indicates SP levels to have significant influence on the integrities of the forceps minor and right CST in the patient group. In contrast, the effect of SP level was not significant for any of the corresponding DTI scalar values in HCs (Fig. 1).

**Table 4:** Results of the regression analyses examining the effect of SP level on WM integrity in medication-naiive patients with MDD and HCs.

	Forceps minor, FA						right CST, RD						right CST, MD					
	Patients with MDD			HCs			Patients with MDD			HCs			Patients with MDD			HCs		
	R^2^		p	R^2^		p	R^2^		p	R^2^		p	R^2^		p	R^2^		p
Model	0.275		0.016	0.165		0.048	0.290		0.011	0.126		0.127	0.286		0.012	0.054		0.571
	R^2^	β	p	R^2^	β	p	R^2^	β	p	R^2^	β	p	R^2^	β	P	R^2^	β	P
Age	0.002	−0.051	0.734	0.015	−0.135	0.334	0.000	−0.019	0.897	0.080	0.310	0.034	0.005	−0.074	0.616	0.037	0.211	0.159
Gender	0.003	0.064	0.694	0.096	−0.447	0.018	0.003	0.058	0.718	0.001	0.050	0.791	0.005	0.084	0.603	0.000	−0.004	0.984
TICV	0.007	0.095	0.549	0.026	−0.243	0.208	0.021	−0.162	0.303	0.002	−0.067	0.734	0.008	−0.102	0.517	0.001	−0.057	0.782
SP	0.247	−0.505	**0.001** ^a^	0.056	−0.244	0.067	0.220	0.476	**0.002** ^a^	0.010	0.104	0.438	0.233	0.490	**0.001** ^a^	0.002	0.044	0.754

R^2^ (coefficient of determination), β (standardized beta) and p values were obtained using linear regression analyses, adjusted for age, gender and total intracranial cavity volume.

^a^Bonferroni correction was applied to control for type I errors: p < 0.016 (0.05/3).

SP, substance p; WM, white matter; FA, fractional anisotropy; RD, radial diffusivity; MD, mean diffusivity; CST, corticospinal tract; MDD, major depressive disorder; HCs, healthy controls; TICV, total intracranial cavity volume.

## Discussion

The present study found significantly lower FA values of the forceps major of the corpus callosum and left ILF, and AD values of the left SLFP in patients with MDD compared to HCs. Also in the patient group, negative correlations were observed between SP levels and FA values of the forceps minor, and positive correlations were observed between SP levels and RD and MD values of the right CST. The effect of SP levels on FA values of the forceps minor and RD and MD values of the right CST, were shown to be significant in patients. The present study is the first report of an association between SP levels and brain WM structural alterations in patients with MDD.

Our results, which showed decreased FA values of the forceps major of the corpus callosum and left ILF, and AD values of the left SLFp, indicate decreased integrity of these regions in patients with MDD, which is in line with the results of previous studies^20, 21^. The corpus callosum is the largest interhemispheric bundle of the human brain, and is an essential component in brain lateralization and inter-hemispheric communication^22^, with critical functions such as emotional processing influenced by this region^23^. The ILF is an association fiber tract that connects the occipital and temporal lobes, including the hippocampus and amygdala^24^, which are the main components of the limbic system related to emotional behavior^25^. The SLF connects the posterior language region with the precentral gyrus and Broca’s regions to form a circuit for higher cortical functions, and alterations in SLF integrity may influence cognitive and language functions which are also impaired in MDD^26^. Therefore, decreased integrity of these regions may predispose individuals to depressive symptoms.

Stress is known to precipitate depressive episodes^27^, and patients with MDD are more likely to have been exposed to stress. SP is released as a part of the stress response^7^, hence SP levels have been shown to be increased when exposed to stress^8^. Stress also has been reported to influence WM integrity^16^, with various WM tracts showing decreased integrity in depression^17^. Therefore, we hypothesized SP levels to show negative associations with WM integrity in patients with MDD, and our results were in line with our initial hypothesis. We observed negative correlations between SP levels and FA values of the forceps minor, and positive correlations between RD and MD values of the right CST in the patient group, which indicate negative associations between SP levels and the integrity of these WM regions in depression. Fibers of the forceps minor interconnect the prefrontal cortex and anterior cingulate areas^28^, and the CST also projects to the anterior cingulate cortex (ACC)^29^. The ACC is linked to both emotional processing and pain circuitry and is known to be activated in response to both physical and emotional pain^30^. The ACC affects spinal nociception through descending modulatory systems as it innervates the periaqueductal gray^31^, which is an important nucleus for endogenous analgesic/antinociceptive systems^32^. Activation of the periaqueductal gray in turn decreases SP levels through the activation of the mu-opioid receptor^33^. As the forceps minor and CST connect the ACC to other structures of the brain, decreased integrity of the forceps minor and CST may influence ACC activity, which may in turn influence SP levels. This may be why we observed correlations between SP levels and the integrity of the forceps minor and CST in particular, among the many other WM tracts. Furthermore, no significant correlations or effects of SP levels on WM integrity were observed in HCs. Although healthy individuals are also exposed to stress, it is unlikely that the stressful situations are chronic such as in depression. Therefore, such individuals are likely to be absent of constant alterations in SP levels, WM integrity and ACC activity, which are conditions often observed in patients with MDD. Our negative results shown in HCs, provide further support for our hypothesis on the associations of SP level and WM integrity in the pathophysiology of depression.

Although initial studies reported NK1 receptor antagonists to have antidepressant effects^3, 4^, subsequent investigations failed to prove the antidepressant efficacy of NK1 receptor antagonists^13^. As currently available antidepressants act on monoamine systems, antidepressant effects of such medications were thought to be brought about by their direct influence on monoamines^34^. However, further downstream molecular events induced by antidepressants have been elucidated, which are similar to the mechanisms of synaptic plasticity, and it is suggested antidepressants exert their effects through enhancing neuroplasticity^35^. Furthermore, WM abnormalities were shown to be pronounced in treatment-resistant depression^20^, and WM integrity has been suggested to play an important role in antidepressant treatment response^36^. However, only the integrity of the forceps minor and CST were correlated with SP levels in our patient group, among the many WM tracts. Although SP itself may be implicated in depression, the lack of antidepressant efficacy of NK1 receptor antagonists may be due to its lack of influence on neuroplasticity, including WM integrity.

To our knowledge, this is the first report on the association between SP and WM structural changes in patients with MDD. As neurotrophic effects of antidepressants have been reported^37^, only medication-naïve patients were included in this study. Although our study has multiple strengths, there are also limitations to consider. Firstly, we relied on a relatively small sample size, and future studies including a larger sample size that replicate our results may be helpful in demonstrating more robust effects. Secondly, we did not assess psychosocial stressors such as childhood adversity and stressful life events. Stress is known to alter SP levels^8^, and WM integrity has been shown to be sensitive to adverse experiences^16^. Therefore, the influence of such environmental factors on our results is uncertain. However, stress is known to precipitate depressive episodes^38^, hence we considered patients more likely to have been exposed to stress compared to HCs. Thirdly, we have measured plasma levels of SP rather than measuring cerebrospinal fluid levels or conducting imaging techniques that can more directly estimate SP levels in the brain. Although numerous studies have reported the role of SP in various disease states by reporting alterations in plasma SP levels^39^, the association between peripheral and central SP levels is still questionable, hence studies that have measured only plasma levels may be considered to be constrained by access to tissue^10^. Nevertheless, previous studies that have attempted to define the association between plasma and cerebrospinal fluid levels of SP, have reported a close correlation between the two^40^. Furthermore, as SP has the ability to pass the blood brain barrier^41^, plasma SP concentration has previously been suggested to be an indirect indicator of SP levels in the central neuronal system^42^. Future studies that apply methods that more directly measure SP neurotransmission in the central nervous system, will further elucidate the conclusions drawn from our results. Fourthly, no significant difference in SP levels were detected between patients with MDD and HCs in our results. This may be due to healthy individuals also being exposed to stress, and SP levels reactively increasing as a stress response^9^, even though such individuals are absent of depressive symptoms. Therefore, the immediate measurement of plasma SP level may be considered limited in representing the long-term changes in SP levels in patients compared to controls. However, our intention was to observe alterations in WM integrity, which is influenced by long-term effects, and not short-term changes in SP levels. We therefore investigated the correlations between SP levels and WM integrity in each diagnostic group separately, and observed correlations in the patient group only as we had initially hypothesized. Lastly, nicotine use was not assessed, which we consider to be a major limitation of our study, as the influence of such a factor on our results is uncertain.

Our study provides evidence for decreased WM integrity in depression, and for the association between SP and WM integrity in MDD. We consider our findings to contribute to neurobiological evidence on the association between SP and brain structural changes in depression, which may be associated with the pathophysiology and treatment of MDD.

## Methods

### Participants

We studied 42 medication-naïve patients with MDD who had never taken antidepressants before, and 57 HCs. Patients were recruited from the outpatient psychiatric clinic of Korea University Anam Hospital, located in Seoul, Republic of Korea. Diagnosis was determined by a psychiatrist according to the Diagnostic and Statistical Manual for Mental Disorders, 4^th^ Edition, Text Revision (DSM-IV-TR), using the Korean version of the Structured Clinical Interview for DSM-IV (SCID-IV). Severity of depression was measured by the 17-item Hamilton Depression Rating Scale (HDRS) on the day of MRI acquisition. Patients with primary or comorbid psychiatric diagnoses other than MDD were excluded from the study. Patients with serious or unstable medical illnesses or primary neurological illnesses, such as cerebrovascular disease, Parkinson’s disease, and epilepsy were also excluded. Fifty-seven HCs were recruited by advertisements in the community. HCs were screened for major psychiatric histories, and none had psychiatric disorders. Subjects with a lifetime exposure to any DSM-IV-TR substance dependence or abuse diagnosis were excluded, with the exception of nicotine. None of the participants had prior or current alcohol use disorders including alcohol abuse or dependence, nor had they been exposed to any kind of substances specified in the DSM-IV-TR substance-related disorders other than nicotine and caffeine. The age of participants ranged from 21–65 years. All participants were right-handed, as revealed by the Edinburgh Handedness Test, and were self-identified Koreans with ethnic origin ascertained by confirming the ethnicity of 3 generations of the patients’ families. The study protocol was approved by the Institutional Review Board of Korea University Anam Hospital in accordance with the Declaration of Helsinki, and informed consent was obtained from all participants.

### Measurement of SP levels

The plasma level of SP was determined by a SP immunoassay Kit (R&D Systems, Minneapolis, USA). Assays were performed according to the manufacturer’s recommendations. Briefly, plasma samples (50 ul) were prepared with a mix of 100 ul calibrator Diluent RD5–45 and 100 ul plasma that were pipetted into each well. 50 uL of the primary antibody solution and SP conjugate were added to each well and incubated at room temperature for 3 hours on a horizontal orbital microplate shaker set at 500 ± 50 rpm. Following a washing procedure, 200 uL of substrate solution (100 uL of the reagent A and B) was added to each well and incubated at 37 °C for 30 minutes. Subsequently, 50 uL of stop solution was added to each well. The optical density of the color reaction was read using a microplate reader at a wavelength of 450 nm. The concentrations of SP in each well were calculated based on a standard curve and the dilution factor. The intra- and inter-assay coefficients of variation for all analyses were less than 8%.

### MRI acquisition

Diffusion data were acquired on a 3.0 T Siemens Trio whole-body imaging system (Siemens Medical Systems, Erlangen, Germany). DTIs were acquired using an echo-planar imaging sequence with the following parameters: repetition time (TR): 6300 ms; echo time (TE): 84 ms; field of view (FOV): 230 mm; 128 × 128 matrix; 3-mm slice thickness with no gap; voxel size 1.8 mm × 1.8 mm × 3.0 mm; diffusion directions = 20; number of slices = 50; b-values: 0 and 600 s/mm2; acceleration factor (iPAT- GRAPPA) = 2 with 38 reference lines for phase encoding direction and 6/8-phase partial Fourier.

### Image processing

The DTIs of the participants were processed using the probabilistic tractography functions in TRACULA (Tracts Constrained by UnderLying Anatomy) implemented in the FreeSurfer 5.3 development version software package (Massachusetts General Hospital, Boston, U.S., http://surfer.nmr.mgh.harvard.edu)^43^. TRACULA determines 18 major WM tracts using an automated method that reconstructs probabilistic distributions of WM pathways from the native DTIs of each participant. Using previously obtained information regarding the likelihood of each WM tract to pass through or next to each cortical parcellation and subcortical segmentation analyzed in the FreeSurfer, TRACULA accurately reconstructs individual WM pathways while preserving the individual variation in WM tracts and assuring selection of the same WM tract in each participant. We performed the TRACULA analysis according to a previously described protocol^44^. We first registered DTIs to the b = 0 images for simple head motion and eddy currents, and then performed registration transformation using FreeSurfer’s bbregister^45^. Using the above transformation, the mapping of cortical parcellation and subcortical segmentation in the DTIs of each participant was reconstructed by FreeSurfer and FSL’s Bayesian Estimation of Diffusion Parameters obtained using sampling techniques^46^. The ball-and-stick model of diffusion was applied to attain each participant’s local diffusion orientations. Probability distributions for 18 major WM tracts were estimated by TRACULA using each participant’s ball-and-stick model and labels of cortical and subcortical segmentation. The 18 major WM tracts include: the forceps major and forceps minor of the corpus callosum, and the anterior thalamic radiation (ATR), cingulum-angular bundle (CAB), cingulum-cingulate gyrus bundle (CCG), corticospinal tract (CST), inferior longitudinal fasciculus (ILF), superior longitudinal fasciculus-parietal bundle (SLFp), superior longitudinal fasciculus-temporal bundle (SLFt), and uncinate fasciculus (UF) in both hemispheres.

Finally, four DTI parameters including fractional anisotropy (FA), axial diffusivity (AD), radial diffusivity (RD), and mean diffusivity (MD) were obtained from delineated individual WM tracts of the participants using FSL’s DTIFit function (http://www.fmrib.ox.ac.uk/fsl). The FA index is the most widely used parameter of DTI, as it is sensitive to the presence and integrity of WM fibers^47^. Higher FA values can represent increases in number and size of axon fibers or decreases in density of crossing fibers^48^, hence AD, RD and MD values have been used to complement FA values in the interpretation of WM microstructure alterations^15^. AD values are considered to be sensitive to axonal damage or degeneration, RD values to be sensitive to demyelination, and MD values to be sensitive to edema, and necrosis^49^. To ensure the quality of the analyses, DTI outputs in all processes were visually inspected by independent researchers (W.S. Tae and J. Kang).

### Statistical Analyses

Differences in demographics between medication-naive patients with MDD and HCs were analyzed using 2-sample t-tests for continuous variables (age and HDRS scores) and a chi-square test for gender and education level. ANCOVA was performed in order to calculate differences in SP levels between the two diagnostic groups (patients with MDD, HCs), with age and gender included as covariates, as both factors have been reported to influence SP levels^9, 50^. ANCOVA was also performed in order to calculate differences in DTI scalar values (FA, AD, RD and MD) for the 18 major WM tracts between the two diagnostic groups, including age, gender and total intracranial cavity volume (TICV) as covariates. To correct for multiple comparisons, Bonferroni correction was applied for the18 WM tracts (p < 0.00278 (0.05/18)).

A 2-tailed Pearson correlation was performed to analyze the correlations between SP levels and DTI scalar values in each diagnostic group, controlling for age, gender and TICV. To correct for multiple comparisons, Bonferroni correction was applied for the18 WM tracts (p < 0.00278 (0.05/18)).

A series of linear regression analyses were performed to examine the effect of SP levels on the integrity of WM tracts that showed significant correlations with SP levels in the correlation analyses. Separate regression models were tested with DTI parameters of the WM tracts as the outcome measures, and SP level as an independent predictor in each group. Each model was adjusted for age, gender, and TICV. To correct for multiple comparisons, Bonferroni correction was applied for the number of values that were significant for the correlation analyses (p < 0.016 (0.05/3)). All statistical analyses were performed using SPSS version 12.0 (SPSS Inc., Chicago, IL, USA).

## Electronic supplementary material

Supplementary information
