# Supplementary material for: The association between substance P and white matter integrity in medication-naive patients with major depressive disorder
Source: Sci Rep. 2017 Aug 29;7:9707. doi: 10.1038/s41598-017-10100-y (PMC5575350; doi:10.1038/s41598-017-10100-y)
Supplement: Supplementary file 1 — Supplementary information [file 41598_2017_10100_MOESM1_ESM.doc]

**The association between substance P and white matter integrity in medication-naive patients with major depressive disorder**

Eunsoo Won^1^, June Kang^2^, Sunyoung Choi ^3^, Aram Kim^2^, Kyu-Man Han^1^, Ho-Kyoung Yoon^1^, Su-Hee Cho^1^, Woo Suk Tae^4^, Min-Soo Lee^1^, Sook-Haeng Joe^1^, Yong-Ku Kim^1*^, and Byung-Joo Ham^1*^

^1^Department of Psychiatry, Korea University College of Medicine, Seoul, Republic of Korea; ^2^Department of Biomedical Science, Korea University, Seoul, Republic of Korea; ^3^Department of Brain and Cognitive Engineering, Korea University, Seoul, Republic of Korea; ^4^Brain Convergence Research Center, Anam Hospital, Korea University Medical Center, Seoul, Republic of Korea;

^*^Corresponding Authors:

Byung-Joo Ham, MD, PhD, Department of Psychiatry, Korea University College of Medicine, Seoul, Republic of Korea. Tel +82-2-920-6843, Fax +82-2-927-2836, E-mail hambj@korea.ac.kr

Yong-Ku Kim, MD, PhD Department of Psychiatry, Korea University College of Medicine, Seoul, Republic of Korea. Tel +82-31-412-5140, Fax +82-31-412-5144, E-mail [yongku@korea.ac.kr](mailto:yongku@korea.ac.kr)

Table S1. FA, AD, RD, and MD values of WM tracts in medication-naiive patients with MDD and HCs.

|  | **WM tracts** | **patients with MDD** | **HCs** | **F** | **p** |
| --- | --- | --- | --- | --- | --- |
| **FA** | **forceps major** | 0.527 (0.0957) | 0.606 (0.0494) | 26.662 | **< 0.001^a^** |
|  | forceps minor | 0.458 (0.0632) | 0.489 (0.0433) | 7.223 | 0.009 |
|  | L ATR | 0.400 (0.0334) | 0.403 (0.0237) | 0.190 | 0.664 |
|  | L CAB | 0.351 (0.0536) | 0.3381 (0.0385) | 3.704 | 0.057 |
|  | L CCG | 0.486 (0.0712) | 0.501 (0.0442) | 1.401 | 0.240 |
|  | L CST | 0.503 (0.0408) | 0.507 (0.0229) | 0.119 | 0.731 |
|  | **L ILF** | 0.465 (0.0471) | 0.497 (0.0320) | 14.371 | **< 0.001 ^a^** |
|  | L SLFP | 0.41 (0.0397) | 0.432 (0.0224) | 8.901 | 0.004 |
|  | L SLFT | 0.452 (0.0451) | 0.470 (0.0225) | 5.872 | 0.017 |
|  | L UNC | 0.407 (0.0407) | 0.418 (0.0300) | 1.777 | 0.186 |
|  | R ATR | 0.397 (0.0308) | 0.403 (0.0244) | 0.639 | 0.426 |
|  | R CAB | 0.360 (0.0609) | 0.361 (0.0355) | 0.046 | 0.831 |
|  | R CCG | 0.466 (0.0603) | 0.480 (0.0427) | 1.695 | 0.196 |
|  | R CST | 0.498 (0.0415) | 0.510 (0.0275) | 2.018 | 0.159 |
|  | R ILF | 0.468 (0.0487) | 0.4950 (0.0334) | 9.727 | 0.002 |
|  | R SLFp | 0.423 (0.0312) | 0.434 (0.0224) | 3.248 | 0.075 |
|  | R SLFt | 0.447 (0.0336) | 0.455 (0.0212) | 1.889 | 0.173 |
|  | R UNC | 0.408 (0.0478) | 0.426 (0.0251) | 5.202 | 0.025 |
| **AD** | forceps major | 0.00150 (0.000150) | 0.00152 (0.0000887) | 0.278 | 0.599 |
|  | forceps minor | 0.00131 (0.0000727) | 0.00133 (0.0000640) | 0.748 | 0.389 |
|  | L ATR | 0.00117 (0.0000714) | 0.00117 (0.0000453) | 0.106 | 0.745 |
|  | L CAB | 0.00129 (0.000215) | 0.00123 (0.0000907) | 3.576 | 0.062 |
|  | L CCG | 0.00126 (0.0000964) | 0.00126 (0.0000694) | 0.000 | 1.000 |
|  | L CST | 0.00128 (0.0000645) | 0.00129 (0.0000439) | 1.294 | 0.258 |
|  | L ILF | 0.00131 (0.0000885) | 0.00135 (0.0000462) | 7.949 | 0.006 |
|  | **L SLFp** | 0.00116 (0.0000579) | 0.00119 (0.0000472) | 12.287 | **0.001^a^** |
|  | L SLFt | 0.00121 (0.0000628) | 0.00124 (0.0000379) | 5.515 | 0.021 |
|  | L UNC | 0.00122 (0.0000558) | 0.00124 (0.0000422) | 5.809 | 0.018 |
|  | R ATR | 0.00117 (0.000117) | 0.00117 (0.0000447) | 0.087 | 0.769 |
|  | R CAB | 0.00130 (0.000180) | 0.00121 (0.00010447) | 10.999 | 0.001 |
|  | R CCG | 0.00119 (0.0000793) | 0.00121 (0.0000633) | 0.821 | 0.367 |
|  | R CST | 0.00129 (0.0000878) | 0.00128 (0.0000506) | 0.247 | 0.620 |
|  | R ILF | 0.00132 (0.0000927) | 0.00135 (0.0000568) | 4.896 | 0.029 |
|  | R SLFp | 0.00115 (0.0000573) | 0.00118 (0.0000525) | 8.954 | 0.004 |
|  | R SLFt | 0.00119 (0.0000602) | 0.00121 (0.0000438) | 1.923 | 0.169 |
|  | R UNC | 0.00121 (0.0000662) | 0.00122 (0.0000464) | 1.584 | 0.211 |
| **RD** | forceps major | 0.000615 (0.000168) | 0.000505 (0.000118) | 13.383 | 0.000 |
|  | forceps minor | 0.000623 (0.000112) | 0.000588 (0.0000621) | 3.714 | 0.057 |
|  | L ATR | 0.000631 (0.0000528) | 0.000623 (0.0000338) | 0.772 | 0.382 |
|  | L CAB | 0.000765 (0.000221) | 0.000743 (0.0000804) | 0.314 | 0.576 |
|  | L CCG | 0.000577 (0.0000880) | 0.000564 (0.0000538) | 0.663 | 0.417 |
|  | L CST | 0.000564 (0.0000600) | 0.000565 (0.0000373) | 0.086 | 0.769 |
|  | L ILF | 0.000620 (0.0000646) | 0.000597 (0.0000365) | 4.249 | 0.042 |
|  | L SLFp | 0.000623 (0.0000547) | 0.000622 (0.0000309) | 0.056 | 0.814 |
|  | L SLFt | 0.000597 (0.0000526) | 0.000594 (0.0000295) | 0.161 | 0.689 |
|  | L UNC | 0.000647 (0.0000563) | 0.000644 (0.0000340) | 0.095 | 0.758 |
|  | R ATR | 0.000642 (0.0000973) | 0.000622 (0.0000321) | 1.797 | 0.183 |
|  | R CAB | 0.000776 (0.000191) | 0.000703 (0.0000913) | 6.384 | 0.013 |
|  | R CCG | 0.000574 (0.0000707) | 0.000568 (0.0000498) | 0.219 | 0.641 |
|  | R CST | 0.000583 (0.0000874) | 0.000558 (0.0000426) | 2.730 | 0.102 |
|  | R ILF | 0.000624 (0.0000696) | 0.000600 (0.0000420) | 4.222 | 0.043 |
|  | R SLFp | 0.000601 (0.0000501) | 0.000606 (0.0000334) | 0.231 | 0.632 |
|  | R SLFt | 0.000589 (0.0000491) | 0.000587 (0.0000314) | 0.077 | 0.782 |
|  | R UNC | 0.000643 (0.0000713) | 0.000624 (0.0000343) | 3.168 | 0.078 |
| **MD** | forceps major | 0.000912 (0.000143) | 0.000844 (0.00010179) | 7.185 | 0.009 |
|  | forceps minor | 0.000854 (0.0000912) | 0.000836 (0.0000514) | 1.740 | 0.190 |
|  | L ATR | 0.000813 (0.0000553) | 0.000807 (0.0000337) | 0.502 | 0.480 |
|  | L CAB | 0.000941 (0.000217) | 0.000907 (0.0000776) | 1.042 | 0.310 |
|  | L CCG | 0.000806 (0.0000723) | 0.000797 (0.0000451) | 0.442 | 0.508 |
|  | L CST | 0.000804 (0.0000546) | 0.000809 (0.0000358) | 0.443 | 0.507 |
|  | L ILF | 0.000852 (0.0000636) | 0.000851 (0.0000303) | 0.042 | 0.837 |
|  | L SLFp | 0.000802 (0.0000506) | 0.000814 (0.0000322) | 1.660 | 0.201 |
|  | L SLFt | 0.000803 (0.0000484) | 0.000809 (0.0000270) | 0.521 | 0.472 |
|  | L UNC | 0.000839 (0.0000511) | 0.000845 (0.0000290) | 0.494 | 0.484 |
|  | R ATR | 0.000821 (0.000102) | 0.000806 (0.0000314) | 0.940 | 0.335 |
|  | R CAB | 0.000953 (0.000185) | 0.000872 (0.0000930) | 8.063 | 0.006 |
|  | R CCG | 0.000782 (0.0000591) | 0.000783 (0.0000406) | 0.000 | 1.000 |
|  | R CST | 0.000820 (0.0000828) | 0.000802 (0.0000406) | 1.764 | 0.187 |
|  | R ILF | 0.000856 (0.0000669) | 0.000853 (0.0000377) | 0.250 | 0.618 |
|  | R SLFp | 0.000784 (0.0000494) | 0.000798 (0.0000363) | 2.417 | 0.123 |
|  | R SLFt | 0.000791 (0.0000478) | 0.000795 (0.0000322) | 0.141 | 0.708 |
|  | R UNC | 0.000832 (0.0000631) | 0.000824 (0.0000334) | 0.693 | 0.407 |

F and p values were obtained using analysis of covariance, adjusted for age, gender and total intracranial cavity volume as covariates.

^a^Bonferroni correction was applied for the 18 WM tracts: 18 comparisons in both hemispheres, p < 0.00278 (0.05/18).

FA, fractional anisotropy; AD, axial diffusivity; RD, radial diffusivity; MD, mean diffusivity; WM, white matter; MDD, major depressive disorder; HCs, healthy controls; L, left hemisphere; R, right hemisphere; ATR, anterior thalamic radiation; CAB, cingulum-angular bundle; CCG, cingulum-cingulate gyrus bundle; CST, corticospinal tract; ILF, inferior longitudinal fasciculus; SLFp, superior longitudinal fasciculus-parietal bundle; SLFt, superior longitudinal fasciculus-temporal bundle; UF, uncinate fasciculus

Table S2. Correlations between SP levels and FA, AD, RD and MD values of WM tracts in medication-naiive patients with MDD

|  | WM tract | FA | AD | RD | MD |
| --- | --- | --- | --- | --- | --- |
|  |  |  |  |  |  |
| SP levels | forceps major | -0.392 (0.014) | -0.033 (0.840) | 0.310 (0.055) | 0.229 (0.161) |
|  | **forceps minor** | **-0.504 (0.001) ^a^** | -0.044 (0.789) | 0.379 (0.017) | 0.298 (0.065) |
|  | L ATR | -0.164 (0.319) | 0.225 (0.168) | 0.272 (0.094) | 0.269 (0.098) |
|  | L CAB | -0.077 (0.643) | -0.139 (0.399) | -0.097 (0.557) | -0.112 (0.498) |
|  | L CCG | -0.297 (0.066) | 0.075 (0.650) | 0.370 (0.020) | 0.331 (0.039) |
|  | L CST | -0.215 (0.188) | 0.208 (0.204) | 0.271 (0.095) | 0.275 (0.090) |
|  | L ILF | -0.318 (0.049) | -0.179 (0.277) | 0.123 (0.456) | 0.007 (0.967) |
|  | L SLFp | -0.391 (0.014) | -0.004 (0.978) | 0.284 (0.079) | 0.205 (0.210) |
|  | L SLFt | -0.420 (0.008) | -0.072 (0.665) | 0.319 (0.048) | 0.207 (0.206) |
|  | L UNC | -0.228 (0.163) | 0.056 (0.734) | 0.206 (0.208) | 0.172 (0.295) |
|  | R ATR | -0.203 (0.214) | 0.188 (0.251) | 0.251 (0.124) | 0.230 (0.158) |
|  | R CAB | -0.166 (0.313) | 0.028 (0.865) | 0.068 (0.683) | 0.056 (0.737) |
|  | R CCG | -0.242 (0.138) | 0.048 (0.770) | 0.220 (0.179) | 0.196 (0.232) |
|  | **R CST** | -0.311 (0.054) | 0.442 (0.005) | **0.486 (0.002) ^a^** | **0.496 (0.001) ^a^** |
|  | R ILF | -0.370 (0.020) | -0.143 (0.384) | 0.243 (0.137) | 0.111 (0.502) |
|  | R SLFp | -0.321 (0.046) | 0.039 (0.813) | 0.228 (0.163) | 0.170 (0.301) |
|  | R SLFt | -0.381 (0.017) | -0.039 (0.816) | 0.261 (0.109) | 0.167 (0.311) |
|  | R UNC | -0.229 (0.161) | 0.145 (0.377) | 0.254 (0.119) | 0.242 (0.138) |

All data are given as coefficient of Pearson correlation controlling for age, gender and total intracranial cavity volume (p value).

^a^Bonferroni correction was applied for the 18 WM tracts: 18 comparisons in both hemispheres, p < 0.00278 (0.05/18).

SP, substance p; FA, fractional anisotropy; AD, axial diffusivity; RD, radial diffusivity; MD, mean diffusivity; WM, white matter; MDD, major depressive disorder; L, left hemisphere; R, right hemisphere; ATR, anterior thalamic radiation; CAB, cingulum-angular bundle; CCG, cingulum-cingulate gyrus bundle; CST, corticospinal tract; ILF, inferior longitudinal fasciculus; SLFp, superior longitudinal fasciculus-parietal bundle; SLFt, superior longitudinal fasciculus-temporal bundle; UF, uncinate fasciculus

Table S3. Correlations between SP levels and FA, AD, RD and MD values of WM tracts in HCs

|  | WM tract | FA | AD | RD | MD |
| --- | --- | --- | --- | --- | --- |
| SP levels | forceps major | -0.012 (.929) | 0.146 (0.294) | 0.065 (0.639) | 0.092 (0.507) |
|  | forceps minor | -0.251 (0.067) | 0.024 (0.862) | 0.250 (0.069) | 0.214 (0.120) |
|  | L ATR | -0.029 (0.838) | 0.011 (0.936) | 0.011 (0.939) | 0.011 (0.934) |
|  | L CAB | -0.003 (0.981) | -0.001 (0.993) | -0.013 (0.928) | -0.009 (0.948) |
|  | L CCG | -0.228 (0.097) | -0.026 (0.854) | 0.195 (0.157) | 0.144 (0.299) |
|  | L CST | -0.030 (0.831) | -0.043 (0.758) | 0.001 (0.997) | -0.017 (0.901) |
|  | L ILF | -0.148 (0.284) | -0.125 (0.368) | 0.127 (0.362) | 0.043 (0.755) |
|  | L SLFp | 0.009 (0.948) | 0.019 (0.892) | -0.038 (0.787) | -0.016 (0.910) |
|  | L SLFt | -0.060 (0.669) | 0.074 (0.594) | 0.074 (0.596) | 0.090 (0.517) |
|  | L UNC | -0.017 (0.906) | 0.002 (0.991) | 0.029 (0.835) | 0.024 (0.863) |
|  | R ATR | -0.094 (0.500) | -0.006 (0.966) | 0.097 (0.485) | 0.063 (0.649) |
|  | R CAB | -0.065 (0.641) | 0.201 (0.146) | 0.188 (0.173) | 0.198 (0.151) |
|  | R CCG | -.0207 (0.133) | 0.069 (0.619) | 0.255 (0.063) | 0.244 (0.075) |
|  | R CST | -.0198 (0.151) | -0.069 (0.620) | 0.108 (0.438) | 0.044 (0.754) |
|  | R ILF | -0.231 (0.093) | 0.058 (0.678) | 0.290 (0.033) | 0.249 (0.069) |
|  | R SLFp | 0.045 (0.745) | 0.077 (0.578) | 0.010 (0.942) | 0.042 (0.763) |
|  | R SLFt | -0.016 (0.908) | 0.099 (0.478) | 0.073 (0.601) | 0.092 (0.507) |
|  | R UNC | -0.142 (0.307) | 0.082 (0.557) | 0.186 (0.177) | 0.165 (0.233) |

All data are given as coefficient of Pearson correlation controlling for age, gender and total intracranial cavity volume (p value).

SP, substance p; FA, fractional anisotropy; AD, axial diffusivity; RD, radial diffusivity; MD, mean diffusivity; WM, white matter; HCs, healthy controls; L, left hemisphere; R, right hemisphere; ATR, anterior thalamic radiation; CAB, cingulum-angular bundle; CCG, cingulum-cingulate gyrus bundle; CST, corticospinal tract; ILF, inferior longitudinal fasciculus; SLFp, superior longitudinal fasciculus-parietal bundle; SLFt, superior longitudinal fasciculus-temporal bundle; UF, uncinate fasciculus
